# P03-15 Activating the ‘peerness' of youth peer leaders in a community sport programme: supporting the development of techne and phronesis in peer leader training

**DOI:** 10.1093/eurpub/ckac095.051

**Published:** 2022-08-29

**Authors:** Julie Hellesøe Christensen, Adam B Evans, Charlotte D Klinker, Marie T Staal, Peter Bentsen, Glen Nielsen

**Affiliations:** 1 Health Promotion Research, Steno Diabetes Center Copenhagen, Herlev, Denmark; 2 Department of Nutrition, Exercise and Sports, University of Copenhagen, Copenhagen, Denmark; 3 GAME, Copenhagen, Denmark; 4 Center for Clinical Research and Prevention, Copenhagen University Hospital - Bispebjerg and Frederiksberg, Copenhagen, Denmark

**Keywords:** Community sport, peer education, peer leader training

## Abstract

**Background:**

Since youth's participation rates in sport drop throughout adolescence, approaches to engage and retain this age group in physical activities are needed. Peers are highly influential on youth's behaviour, and peer education is widely used for youth health promotion purposes to harness this peer influence. Peer education builds on the rationale that youth who share characteristics that identify them as peers will have increased credibility, identification, and role modelling in their interactions. However, it is often unclear how the rationales of peer education are activated in peer-led programmes. We therefore asked: How can peer leaders be supported in activating their? peerness' in health promotion programmes?

**Methods:**

The study was conducted in a non-profit organisation, GAME, where youth (age 16-25) are trained to lead street sport activities for younger children (age 8-15) in low resource neighbourhoods. The youth leaders were often locals and thus shared a peer relation with the participating children based on age and growing up in the same community. The empirical material consisted of learning objectives for GAME's peer leader training programme and field notes from observations (approximately 54 hours) of the peer leader training. To explore educational content that can support the rationales of peer education, the qualitative analysis drew on contemporary interpretations of two forms of knowledge;techne (practical knowledge) and phronesis (practical wisdom), originally proposed by Aristotle.

**Results:**

Techne was supported in training activities that prepared peer leaders for making decisions related to the implementation of street sport activities while taking contextual factors (e.g. the weather or the participants' experience) into consideration. Phronesis was supported in training activities that encouraged peer leaders to reflect on their position and leadership approach and to support positive social relations among the participants. Training that supported phronesis encouraged experience-based critical reflection and decision-making rather than providing a recipe of how to act.

**Conclusion:**

Supporting phronesis in peer leader training will encourage peer leaders to use their peerness actively. To meet the rationales of peer education, peer leaders' practical wisdom should be acknowledged as a key component in peer leadership and this should be reflected in peer leader training.

